# Diarrhea or No Diarrhea, It Still Hurts: An Atypical Case of Clostridioides difficile

**DOI:** 10.7759/cureus.9900

**Published:** 2020-08-20

**Authors:** Abhinav Singla, David Pash

**Affiliations:** 1 Department of Internal Medicine , Pacific Northwest University of Health Sciences, Yakima, USA; 2 Internal Medicine, Skagit Valley Hospital, Mount Vernon, USA; 3 College of Osteopathic Medicine, Pacific Northwest University of Health Sciences, Yakima, USA

**Keywords:** clostridium difficle infection, abdominal pain without diarrhea

## Abstract

Clostridioides difficile (C. difficile) is a common nosocomial infection that is classically described as profuse watery diarrhea in hospitalized patients after antibiotic use. We present a case of a 76-year-old female who presented to our emergency room with diffuse abdominal pain after consuming meals. This patient had completed treatment with oral vancomycin for C. difficile infection two weeks prior to admission and had been asymptomatic until this point. After receiving treatments for presumed acute mesenteric ischemia did not yield clinical improvement, polymerase chain reaction for C. difficile stool antigen was tested and was positive. While the patient did not have diarrhea, the classical feature of C. difficile infection, she quickly improved after treatment with oral vancomycin.

## Introduction

Clostridioides difficile (formally Clostridium difficile; C. difficile) is a common cause of nosocomial diarrhea that is commonly seen after the termination of antibiotic therapy. The antibiotics with the highest incidence of Clostridium difficile infection (CDI) are fluoroquinolones, clindamycin, penicillins, and cephalosporins [1]. Other risk factors for CDI include age >65 years, proton pump inhibitor use, hospitalization, inflammatory bowel disease, and other comorbid illnesses [2]. CDI is a significant cause of in-hospital mortality; in 2017, there were an estimated 224,000 cases of CDI in hospitalized patients, and it attributed to approximately 12,800 deaths [3].

Transmission of C. difficile is via the fecal-oral route after ingestion of spores that can be found on the hands of health care workers and common sites within hospitals such as commodes, tubs, bed rails/linens, etc. [4,5]. It is well established that the use of hand sanitizers and other alcohol-based rubs is insufficient to kill C. difficile spores, and therefore, handwashing is required for all medical personnel to minimize transmission risk [6].

Upon disruption of normal intestinal flora from antibiotic usage, C. difficile is able to germinate and cause a variety of clinical manifestations. The main clinical symptom of CDI is profuse watery diarrhea. Other clinical features include abdominal pain/cramping, nausea, vomiting, and anorexia.

Diagnosis of CDI is confirmed via positive laboratory stool testing of C. difficile toxins or C. difficile toxin B gene. Laboratory testing, however, is reserved for patients who have acute diarrhea (more than three loose/unformed stools within 24 hours) without any other identifiable cause [7,8].

We present an interesting clinical case of an uncommon presentation of a suspected recurrent CDI in a female that presented with profuse abdominal pain without diarrhea.

## Case presentation

A 76-year-old female presented to the emergency room after a two-day history of diffuse abdominal pain, nausea, dry heaving, anorexia, and generalized weakness. The abdominal pain became worse with eating and was relieved while lying supine and not consuming meals. She denied having diarrhea or any loose stools on the presentation or within the last two weeks prior to her arrival in the emergency room. She had a past medical history of hypertension, hyperlipidemia, paroxysmal atrial fibrillation, type 2 diabetes, and end-stage renal disease that was requiring hemodialysis three times per week. Approximately two months prior, she had been hospitalized for methicillin-resistant Staphylococcus epidermidis bacteremia that was suspected to be related to her dialysis catheter. After release from the hospital, she developed acute diarrhea and was positive for CDI and was treated with oral vancomycin for 10 days. By the time she presented to our emergency department, she had not had diarrhea since the completion of her vancomycin treatment that had been completed two weeks prior.

**Figure 1:**
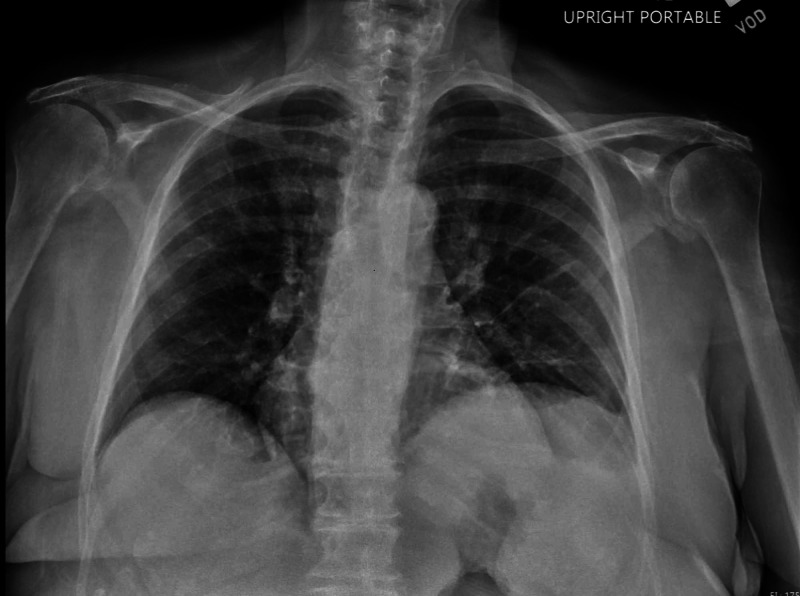
Chest X-Ray (anterior-posterior view) No cardiopulmonary pathology.

On examination, she was alert and oriented however, in moderate distress secondary to her abdominal pain. She was afebrile, hypertensive, and hemodynamically stable. Her abdominal exam found hypoactive bowel sounds in all quadrants, mild distension, and was diffusely tender. The rest of her physical exam was normal. Initial laboratory studies showed a leukocyte count of 7.0 x 10^3/uL (normal range: 4-11 x 10^3/uL), hemoglobin 8.9 g/dL (normal range: 12.0-15.5 g/dL), hematocrit 29.5% (normal range: 34.9%-44.5%) and platelets 100 x 10^3/uL (normal range: 150-450 x 10^3/uL). Renal function tests showed a BUN of 38.0 mg/dL (normal range: 8-20 mg/dL) and creatinine of 2.90 mg/dL (normal range: 0.6-1.5 mg/dL). Liver function tests were normal. The chest X-ray in the emergency department showed no acute cardiopulmonary processes (Figure 1).

**Figure 2:**
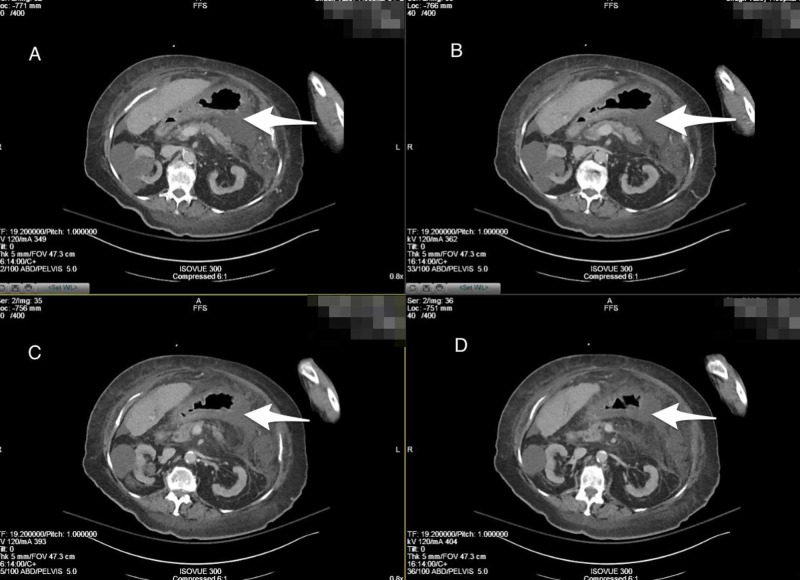
CT Abdomen (axial plane) A-D) Cross-sectional view of abdomen showing fluid around the gastric area.

CT scan of the abdomen was initially concerning for a perforated viscous as there was evidence of fluid around the stomach; however, consultation with surgery determined that this was more characteristic of enteritis and not due to perforation (Figure 2).

Due to her recent history of CDI, a C. difficile enzyme immunoassay for glutamate dehydrogenase toxin and C. difficile polymerase chain reaction (PCR) for toxins A+B were performed on a stool sample and were both positive.

**Figure 3:**
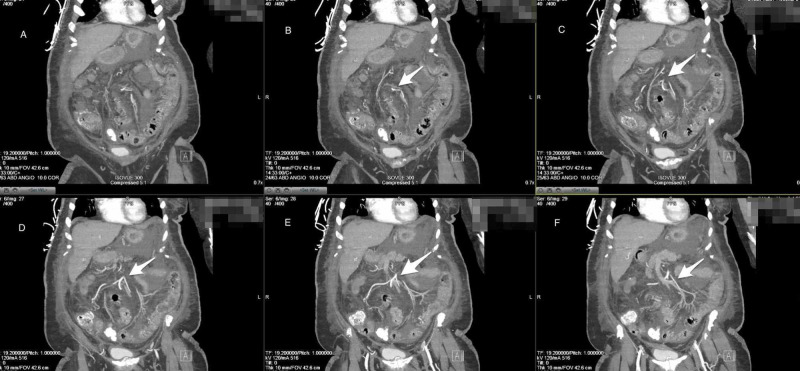
CT Angiography Abdomen and Pelvis (coronal plane) A-F) Showing no filling defects in the mesenteric arteries and only moderate atherosclerotic plaque formation of the same vessels

Acute/chronic mesenteric ischemia was suspected in this patient due to her past medical history and her presentation with an acute abdomen that was subjectively worsened with meals. CT angiography, however, determined that there were no filling defects in the mesenteric arteries and only moderate atherosclerotic plaque formation of the same vessels (Figure 3).

An upper endoscopy was performed with biopsies of the gastric mucosa that were negative for gastritis and dysplasia.

The patient was initially treated with bowel rest and IV fluid resuscitation; however, her symptoms persisted and were unchanged for two days. The patient was started on oral vancomycin 125 mg on the presumption of an atypical presentation of CDI after consultation with a gastroenterologist. The patient improved her clinical status after two days of antibiotics. She was discharged on day eight of her hospitalization; at this time, she was no longer symptomatic.

## Discussion

We presented a case of a female with an uncommon presentation of CDI without diarrhea and abdominal pain. The patient also presented with enteritis, which is also atypical of C. difficile gastrointestinal infections [9,10]. The need for early diagnosis of CDI is important because of the high mortality rate associated with infection. In 2011, there were 450,000 CDI cases in the United States that resulted in 29,300 associated deaths [6]. Approximately 20% of hospitalized patients are carriers of C. difficile, and up to 50% of nursing home residents may be carriers as well [11]. These populations are the most susceptible to colonization and subsequent infection with C. difficile. Recent efforts and initiatives for antibiotic stewardship have resulted in the reduction in C. difficile transmission in hospitals that have reduced the amount of CDI associated deaths [12]. Further reduction via the establishment of early diagnosis and treatment is necessary to prevent further CDI complications and death.

Apart from several case reports, documentation of CDI without diarrhea is not well established in the literature. The literature and clinical recommendations, including from the Infectious Disease Society of America (IDSA), are that the diagnosis of CDI is primarily established with the observation of the clinical signs and symptoms of CDI (more than three watery diarrhea in a 24-hour period), and is then confirmed with laboratory testing [7,8,13,14]. Curiously, these recommendations from the IDSA are considered to be based on a “very low quality of evidence” [8].

## Conclusions

Regardless of advancements in treatment, mortality from CDI still remains high. It is unclear how many patients may have CDI without diarrhea. Our case shows that a high index of suspicion is important in establishing a diagnosis of CDI with an atypical presentation. There is a need for future studies to examine the prevalence of CDI without diarrhea and the risk factors that may lead to this presentation. Additional clarification regarding the diagnostic criteria of CDI should include atypical presentations to avoid delayed or missed diagnoses.
